# First report of cutaneous leishmaniasis caused by *Leishmania donovani* in Ethiopia

**DOI:** 10.1186/s13071-023-06057-9

**Published:** 2023-12-16

**Authors:** Gashaw Azanaw Amare, Gebeyaw Getnet Mekonnen, Mekibib Kassa, Ayenew Addisu, Desalegn Adane Kendie, Banchamlak Tegegne, Adugna Abera, Dagimawie Tadesse, Seid Getahun, Yenesew Mihret Wondmagegn, Behailu Merdekios, Mezgebu Silamsaw Asres, Johan van Griensven, Gert Van der Auwera, Saskia van Henten, Myrthe Pareyn

**Affiliations:** 1 Debre Markos University, Debre Markos, Ethiopia; 2 University of Gondar, Gondar, Ethiopia; 3 Amhara Public Health Institute, Bahir Dar, Ethiopia; 4 Ethiopian Public Health Institute, Addis Ababa, Ethiopia; 5 Arba Minch University, Arba Minch, Ethiopia; 6 Wollo University, Dessie, Ethiopia; 7 Institute of Tropical Medicine Antwerp, Antwerp, Belgium

**Keywords:** Cutaneous leishmaniasis, Species typing, Ethiopia, *Leishmania donovani*, *Leishmania aethiopica*

## Abstract

**Background:**

Leishmaniasis is a common neglected tropical disease in Ethiopia. Visceral leishmaniasis (VL) caused by *Leishmania donovani* presents in the lowlands, while cutaneous leishmaniasis (CL) affects people living in the highlands. Although CL is described as being caused by *Leishmania aethiopica*, there is also evidence of *L. tropica* and *L. major* isolated from a patient, sand flies and potential reservoirs. Information on species causing CL in Ethiopia is patchy, and no nation-wide study has ever been done. Understanding which species are causing CL in Ethiopia can have important implications for patient management and disease prevention.

**Methods:**

We analyzed stored routine samples and biobanked DNA isolates from previously conducted studies of CL patients from different centers in the north, center and south of Ethiopia. Species typing was performed using ITS-1 PCR with high-resolution melt (HRM) analysis, followed by HSP70 amplicon sequencing on a selection of the samples. Additionally, sociodemographic, clinical and laboratory data of patients were analyzed.

**Results:**

Of the 226 CL samples collected, the *Leishmania* species could be determined for 105 (45.5%). *Leishmania aethiopica* was identified in 101 (96.2%) samples from across the country. In four samples originating from Amhara region, northwestern Ethiopia, *L. donovani* was identified by ITS-1 HRM PCR, of which two were confirmed with HSP70 sequences. While none of these four patients had symptoms of VL, two originated from known VL endemic areas.

**Conclusions:**

The majority of CL was caused by *L. aethiopica*, but CL due to *L. tropica* and *L. major* cannot be ruled out. Our study is the first to our knowledge to demonstrate CL patients caused by *L. donovani* in Ethiopia. This should spark future research to investigate where, how and to which extent such transmission takes place, how it differs genetically from *L. donovani* causing VL and whether such patients can be diagnosed and treated successfully with the currently available tools and drugs.

**Graphical Abstract:**

**Figure:**
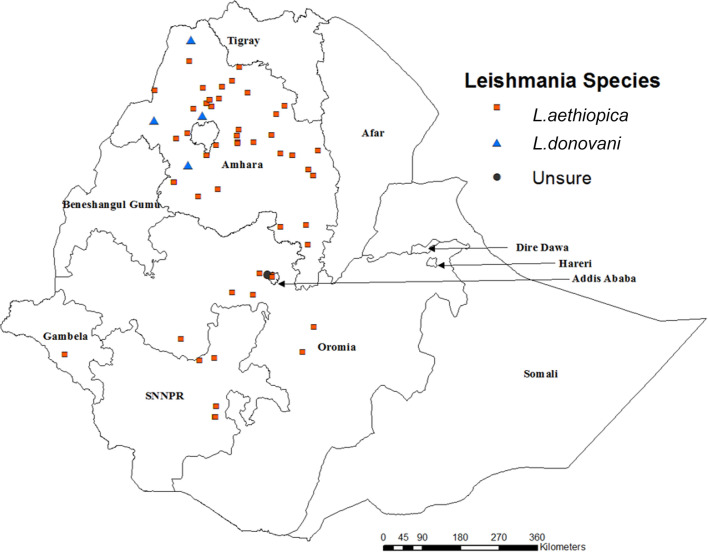

## Background

Leishmaniasis is a neglected tropical disease (NTD) caused by *Leishmania* protozoa. Depending on the parasite species, it can manifest as cutaneous (CL) and visceral (VL) leishmaniasis, which both pose an important public health concern in Ethiopia [1]. VL is caused by *Leishmania donovani* and is prevalent in the lowlands in the northwest and south of the country, where *Phlebotomus orientalis* and *P. martini* are the vectors, respectively [2]. Annually, 2000 to 4000 VL patients are reported [1]. CL is predominantly caused by *Leishmania aethiopica*, which is transmitted by *Phlebotomus longipes* and *P. pedifer* [3, 4]. It mainly occurs in the highlands bordering the Great Ethiopian Rift Valley, ranging from northwest to southwest and south to east Ethiopia [5]. CL is the most common form, with an estimated 20,000 to 50,000 cases annually [1]. CL in Ethiopia has extremely heterogenous presentations, with few lesions presenting as typical *Leishmania* ulcers. Lesions are usually classified as localized CL (LCL), mucocutaneous leishmaniasis (MCL) or diffuse CL (DCL). CL caused by *L. aethiopica* is generally severe, chronic and predominantly occurs on the face [6, 7].

*Leishmania aethiopica* is considered the causative agent of CL in Ethiopia, although there is some evidence indicating that other species are potentially involved. Both *Leishmania tropica* and *L. major*, the CL-causing *Leishmania* species in northern Africa, have been found in Ethiopia in animals (e.g. bats, rodents) [8, 9] and sand flies [10, 11], which could potentially be disease reservoirs and vectors respectively. Only few clinical studies have been performed in which the *Leishmania* species causing CL was determined [12–14]. Among the samples tested, all except one showed that the species causing CL was *L. aethiopica*. This single report was a CL case from the Awash Valley in eastern Ethiopia, which was caused by *L. tropica* [14]. Most of these studies were done in central and southern Ethiopia, where a small number of clinical samples were typed. Moreover, these studies were limited to conventional methods like PCR restriction fragment length polymorphism (RFLP) and iso-enzyme typing.

Understanding which species are causing CL in the country is important to understand the transmission dynamics and different clinical presentations of CL but could also have implications for the use of diagnostics and treatment regimens. Therefore, this study aimed to assess the *Leishmania* species causing CL in Ethiopia using samples from different treatment centers in the country.

## Methods

### Study samples

**Fig. 1:**
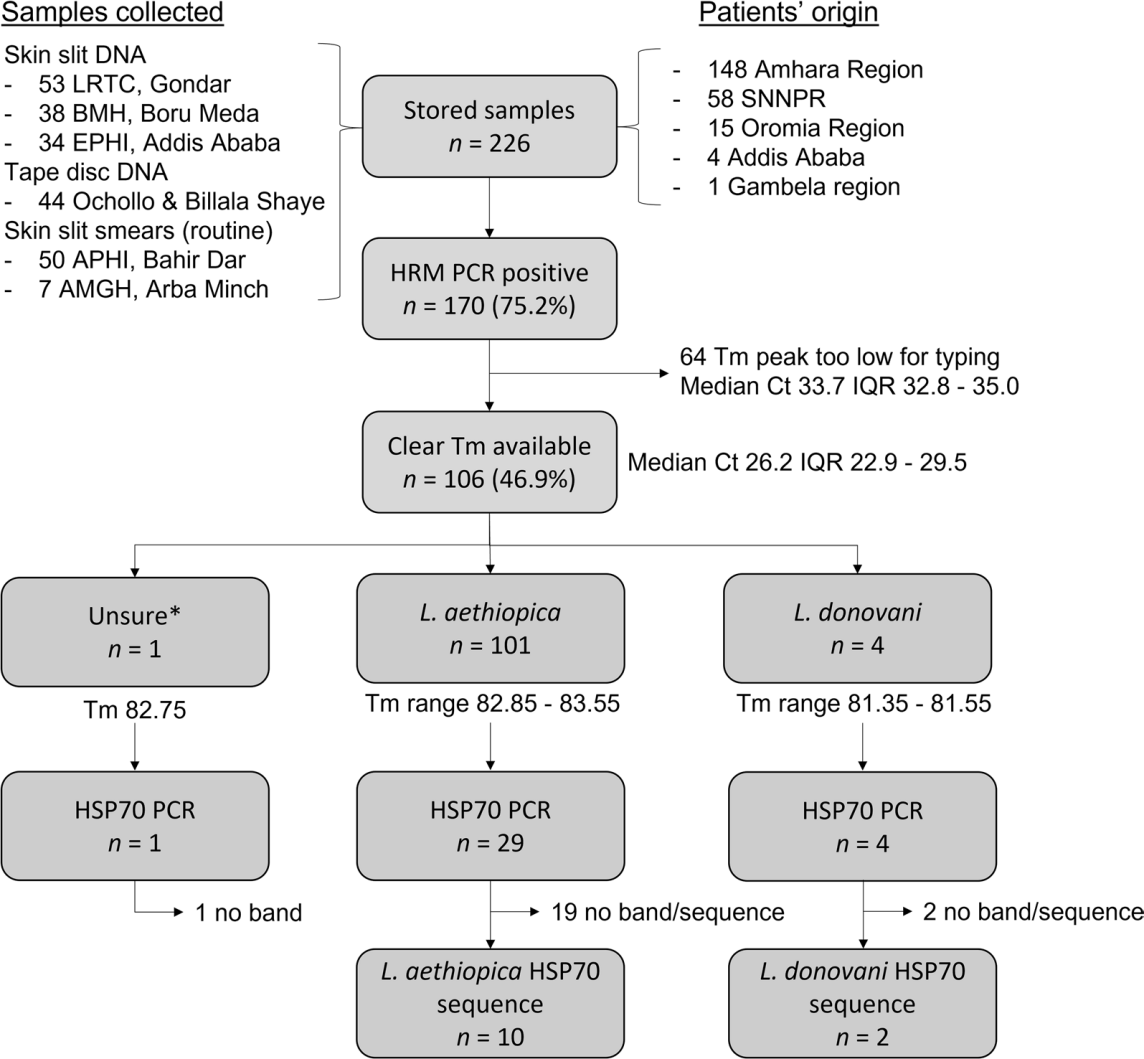
Overview of species typing tests performed on samples derived from different geographical regions in Ethiopia. *LRTC* Leishmaniasis Research and Treatment Center, *APHI* Amhara Public Health Institute, *BMH* Boru Meda Hospital, *EPHI* Ethiopian Public Health Institute, *AMGH* Arba Minch General Hospital, *SNNPR* Southern Nations Nationalities and Peoples’ Region, *HRM PCR* high-resolution melt PCR, *Tm* melting temperature, *IQR* interquartile range, *HSP70* heat-shock protein 70. *Sample could not be unambiguously identified based on ITS-1 Tm

This study was performed on 226 stored samples collected from CL patients at leishmaniasis treatment facilities in Ethiopia (Fig. 1). Twelve major treatment centers were contacted to ask whether they kept stored routine slides or study samples from CL patients, of which five indicated they wanted to participate in the study, leading to a total of 226 stored samples. Routine Giemsa-stained *Leishmania*-positive slit-skin smears were collected from Arba Minch General Hospital (AMGH, *n* = 7) in the south and Amhara Public Health Institute (APHI, *n* = 50) in Bahir Dar, northwest Ethiopia. Additionally, bio-banked DNA extracts of slit-skin and tape disc samples (isolation performed with commercial extraction kits) were employed from previous studies conducted on CL at Ethiopian Public Health Institute (EPHI, *n* = 34 slit-skin DNA) in Addis Ababa, Boru Meda Hospital (BMH, *n* = 38, slit-skin DNA) in the northeast [15], Leishmaniasis Research and Treatment Center at the University of Gondar (LRTC, *n* = 53 slit-skin DNA) in the northwest [16] and Ochollo and Billala Shaye villages (*n* = 44, tape disc DNA) in the south. From these studies, we selected samples that were previously positive by kinetoplast DNA (kDNA) qPCR. Species typing was not performed in any of these studies, except for five samples from the research executed in Boru Meda [15], which are not included in this study and were all *L. aethiopica*.

Basic information such as the district where the patient came from and the type of CL were collected from previous study documents and laboratory registration forms. Additionally, from *L. donovani* CL patients, more detailed information was collected on the clinical characteristics of their lesion from the chart. If lesion pictures were available from previous studies, these were collected as well. If VL signs or symptoms (e.g. fever, enlarged vital organs, pancytopenia, rK39 rapid diagnostic test results) and HIV status were assessed and described in the patient chart, this was recorded as well.

### Molecular analysis

Slit-skin samples and DNA isolates from other sites were shipped (DNA in cold chain) to LRTC and stored at room temperature and − 80 °C respectively until further analysis. All analyses were conducted at the LRTC molecular laboratory at the University of Gondar Hospital in northern Ethiopia.

#### DNA extraction

DNA was extracted from the slit-skin smears collected from AMGH and APHI using the Maxwell 16 LEV Blood DNA extraction kit (Promega, Leiden, The Netherlands) following the manufacturer’s instructions. Briefly, 30 µl lysis buffer was added and rubbed on the microscopy slide, and the material on the slide was transferred to a new vial. The remaining volumes of lysis buffer and Proteinase K were added, and samples were incubated at 56 °C for 20 min at 400 rpm. Samples were transferred to the automatic Maxwell 16 Instrument (AS1000, Promega) and processed according to the manufacturer’s instructions. Elution was done in 50 µl nuclease-free water, after which the DNA was stored at –20 °C for further analysis. In each extraction batch of 15 samples, one negative extraction control (lysis buffer only) was included.

#### ITS-1 high-resolution Melt (HRM) PCR

All DNA extracts (bio-banked from previous studies and extracted for the purpose of this study) were subjected to a real-time PCR followed by a high-resolution melt curve (HRM) analysis for species typing based on the 265–288 bp (depending on species) ITS-1 region of the leishmanial ribosomal RNA operon using the ITS-219F and ITS-219R primers as described by Talmi-Frank et al. [17]. DNA of cultivated *Leishmania* species (*L. aethiopica* MHOM/ET/72/L127, *L. tropica* MHOM/PS/2003/LRC-L1022*, L. tropica* MHOM/IL/2002/LRC-L863, *L. major* Githure (Kenya)*, L. donovani* MHOM/SD/2007/Ged4) were used as positive controls in each run. Additionally, a non-template control (nuclease-free water) and the negative extraction controls were included. The HRM PCR results were considered positive when the derivative of the fluorescence of the melting temperature (Tm) curve exceeded 1 unit and the Tm of the sample was similar to the Tm of the positive control of a certain species and considerably distinct from the Tm of other *Leishmania* species in that run.

#### HSP70 sequencing

Samples that met the criteria for the height of the melting curve, but which were at the outer ranges of Tm values for a species, or for which the species could not be unambiguously identified, were subjected to a conventional PCR targeting the 1245-bp F fragment of the HSP70 gene using HSP70-F25 and HSP70-R1310 primers as described by Van der Auwera et al. [18] and visualized on a 1.5% agarose gel. If no band was observed, a nested PCR was conducted on the PCR product to amplify the overlapping 522-bp N fragment and 723-bp T fragment of the HSP70 gene separately using HSP70-4F and HSP70-4R primers for the N fragment and HSP70-7F and HSP70-6R primers for the T fragment. If amplification was successful, PCR products were cleaned up and subjected to amplicon sequencing using the sequencing primers in [18].

Dendrograms were built in the MEGA package version 7 [19]. Reference sequences were included from *Leishmania* isolates of relevant species including *L. donovani, L. tropica, L. aethiopica, L. major*, *L. infantum*, and the *L. mexicana* species complex as outgroup. Neighbor-joining trees were constructed based on p-distances, using 2000 bootstrap samples.

## Results

### Study population

The 226 samples used in this study were derived from 98 districts in five regions in the country, namely 148 from Amhara Region, 58 from the Southern Nations, Nationalities and Peoples’ Region (SNNPR), 15 from Oromia Region, 4 from Addis Ababa and 1 from Gambela Region (Fig. 1). The clinical form of CL was recorded for 219 samples, with most patients presenting with LCL (*n* = 100, 45.7%) and 98 (44.7%) with MCL and 21 (9.6%) with DCL, although definitions may differ between sites.

### *Leishmania* species typing

Overall, 170 (75.2%) samples were positive by HRM PCR, but Tm peaks only met the criteria to identify the species of *Leishmania* for 106 out of 226 (46.9%) samples (Fig. 1). These samples had a median Ct value of 26.6 (IQR 22.9—29.5), which is considerably lower than Ct values of the samples for which the species could not be determined (median Ct 33.7, IQR 32.8 – 35.0). *Leishmania aethiopica* was identified as the causing species of CL for 101 out of 106 (95.3%) samples, with broad Tm’s ranging between 82.85 °C and 83.55 °C. Four samples (3.8%) were identified as *L. donovani*, two with a Tm of 81.35 °C and two 81.55 °C (Fig. 1). For one sample, with a Tm of 82.75 °C, the species could not be unambiguously identified.

**Fig. 2:**
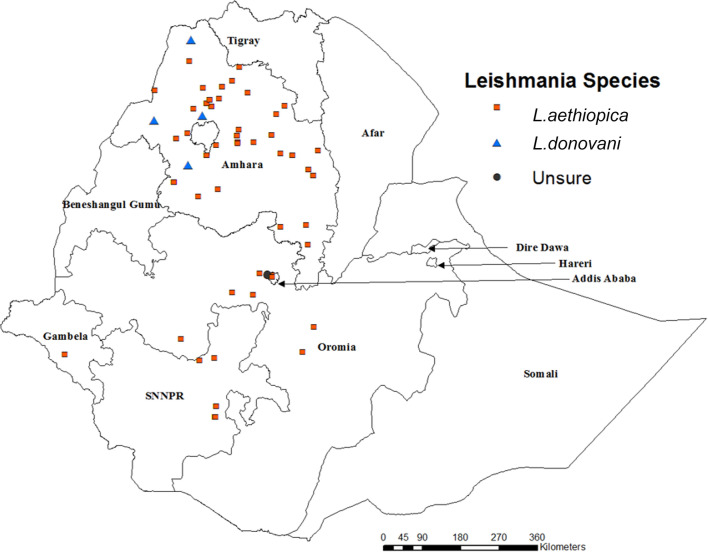
Geographical distribution of CL patients’ samples and the causing *Leishmania* species in Ethiopia [39, 40]

Of the 101 samples identified as *L. aethiopica*, 76 were from Amhara Region, 18 from SNNPR, five from Oromia Region, and one each from Addis Ababa and Gambela Region, clearly depicting the mountain range of the Ethiopian Great Rift Valley in Ethiopia (Fig. 2). All four *L. donovani* samples originated from patients from Amhara Region in northwest Ethiopia.

**Fig. 3:**
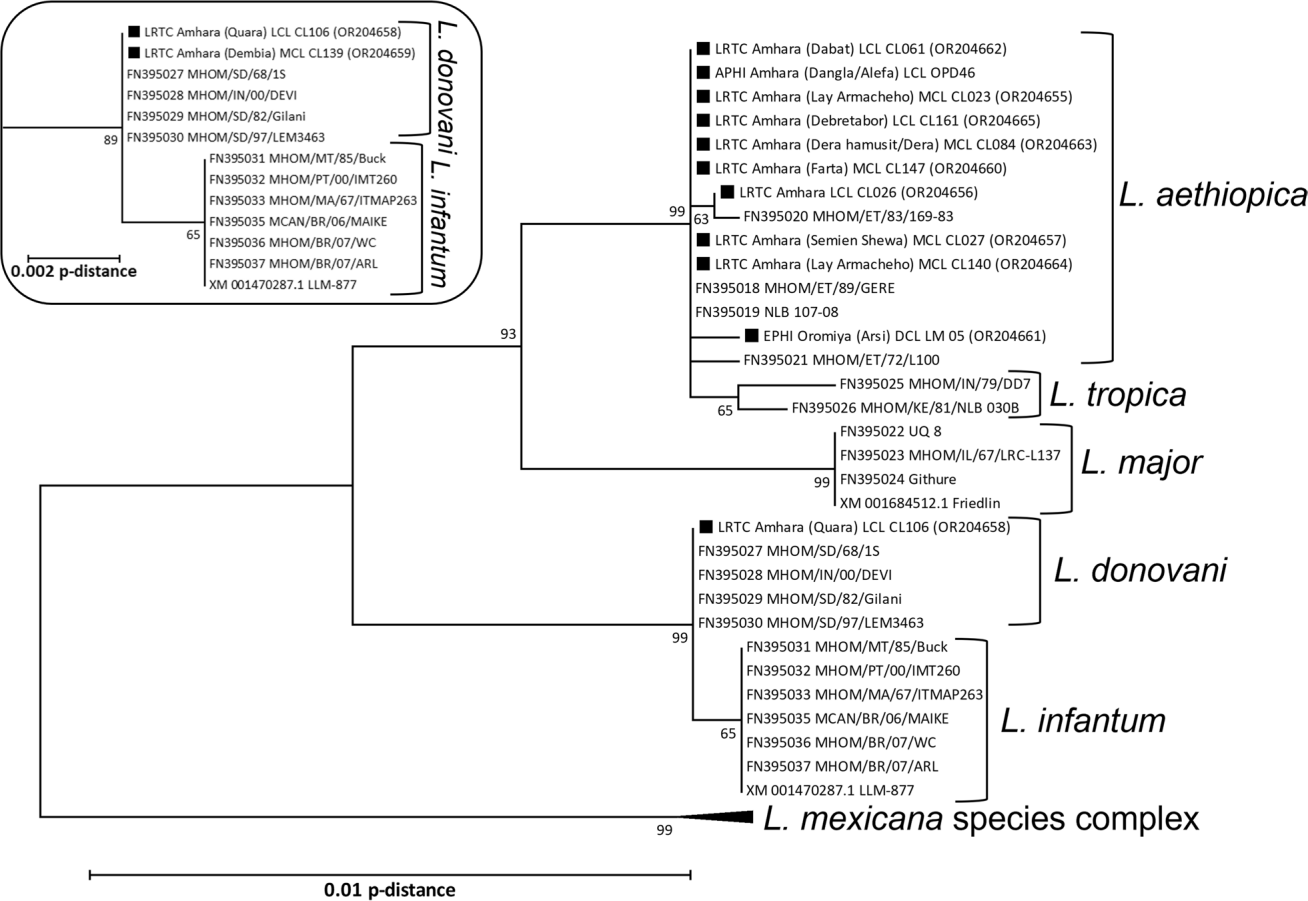
HSP70 sequence analysis of *Leishmania* strains included in this study compared against reference sequences from GenBank. Strains of this study are identified with a black square, followed by the strain name, region where it was found (district or health facility in parenthesis), clinical form (abbreviations see main text), sample ID and GenBank accession number in parentheses. Reference sequences are identified with their GenBank accession followed by the strain name. Sequences of the *Leishmania mexicana* species complex were used as outgroup. The main dendrogram was constructed based on a partial 1245-bp fragment of the coding sequence (fragment F in [18]). For one additional strain only the 552-bp N fragment could be determined, and the *Leishmania donovani* species complex from the corresponding analysis is shown in the top left insert. Bootstrap support is depicted in percentages at the nodes if > 50%. The distance scale is shown at the bottom left

Of the 34 strains included in this study for which HSP70 PCR was attempted, sequencing was successful only in 12 cases (Fig. 3). Ten of these confirmed the ITS-1 *L. aethiopica* typing, and two confirmed the ITS-1 *L. donovani* typing. The remaining 22 ITS-1 typing results could not be confirmed. This included the sample that could not be unambiguously identified based on the HRM PCR with a Tm of 82.75 °C. Sequences were deposited at GenBank under accession numbers OR204655 to OR204665, with the two *L. donovani* sequences identified as MHOM/ET/2021/CL161 and MHOM/ET/2020/CL139.

### Clinical description of CL caused by *L. donovani*

**Fig. 4:**
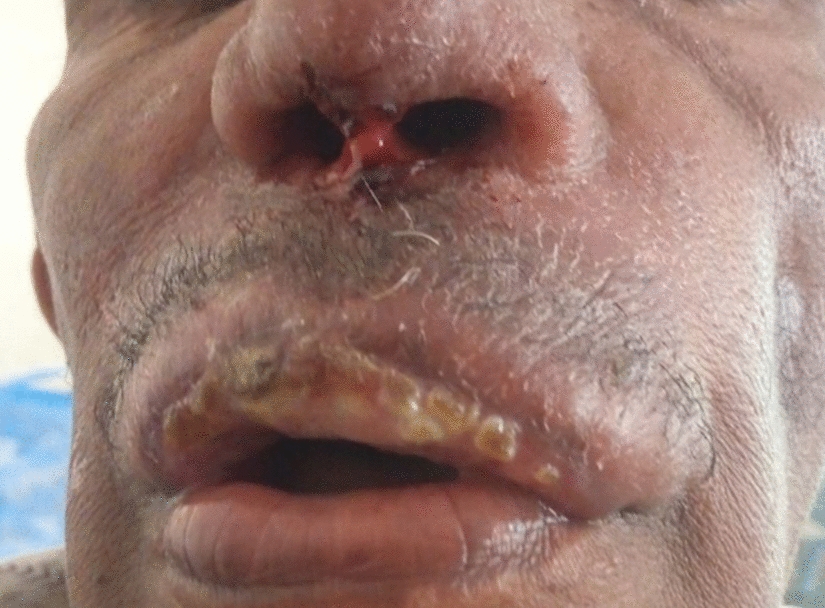
Mucocutaneous lesion caused by *Leishmania donovani* in a patient from Kafta Humera district, northern Ethiopia. The lesion involved the patient’s mouth and nose (nasal septum) and showed erythematous swelling with ulceration and signs of superinfection

The first *L. donovani* CL patient was a 56-year-old male who originated from Kafta Humera in northern Ethiopia (Fig. 2), which is endemic for VL [20, 21]. He presented with a 4-year-old lesion, classified as MCL. The lesion was on the patient’s mouth and nose, showed erythematous swelling with ulceration and signs of superinfection and involved the nasal septum (Fig. 4). The patient was HIV negative, presented with normal vital signs without fever and had no history of VL. While the slit-skin microscopy was negative, the patient had a positive kDNA PCR result on slit skin with Ct 20.7 in the study from which the sample was employed [16]. Although the patient was started on systemic treatment with sodium stibogluconate (SSG), it had to be discontinued because of hypokalemia. One year later, the patient returned because of lesion worsening and was referred to Abdurafi Health Center for treatment with SSG and miltefosine; treatment outcomes are not available.

Another 28-year-old male *L. donovani* CL patient came from Quara, situated between 500 and 1800 m altitude in western Gondar and also known to be VL endemic [22]. The patient was classified as LCL and presented with four lesions of 6-month duration, of which the largest was 5 × 4 cm, occurring on the nose, and multiple erythematous lesions on the lip and cheeks. The patient was negative for HIV, had no systemic symptoms or fever, and vital organs and complete blood counts were normal. The routine slit-skin microscopy for this patient was also negative, but kDNA PCR on the slit-skin sample was positive with a Ct of 18.3 in [16]. Although there was improvement after 28 days of SSG treatment, the patient returned with worsening of the index lesion and manifestation of new papules on the forehead. After improvement with another cycle of SSG, he did not return to the LRTC anymore and outcomes are unavailable.

Of the other two *L. donovani* CL cases, no detailed information could be retrieved. One patient was a 25-year-old male who had lived his whole life in Durbete at an altitude ranging between 1200 and 2000 m. He presented with a plaque lesion with 2 × 1-cm papules of 18-month duration on the nasal tip. The patient was negative for HIV as well as slit-skin microscopy for *Leishmania* yet positive by kDNA PCR on slit skin with a Ct of 27.4. The other patient was 25 years old and had lived his whole life in Dembia, which lies at around 1800 m. He was classified as DCL and presented with nine lesions of 24-month duration limited to the face. The lesions were nodular, crusted, swollen, exfoliative, erythematous and hypopigmented. The patient’s HIV status was unknown, but he was positive for *Leishmania* microscopy with a grading of + 2 and showed a positive kDNA PCR on slit skin with a Ct of 21.4, also in [16].

## Discussion

Our study is the first to conduct country-wide *Leishmania* species typing for CL patients and to demonstrate that *L. donovani* can cause CL in Ethiopia. This was confirmed by ITS-1 HRM PCR and HSP70 amplicon sequencing.

CL due to an infection with *L. donovani* has been reported in Sri Lanka as well, where CL-causing *L. donovani* strains were shown to be genetically different from *L. donovani* causing VL in its neighboring countries [23, 24]. In Kenya and Sudan, where CL is normally caused by *L. tropica* or *L. major*, isolates of CL patients were confirmed to be *L. donovani* by isoenzyme typing and PCR-RFLP [25, 26]. While no further clinical description was available for the cases from Kenya, patients in Sudan presented with typical CL lesions and had no travel history to a VL-endemic region or symptoms of VL or PKDL [26].

The four *L. donovani* CL cases in this study manifested in all three clinical forms (LCL, DCL and MCL). While atypical presentations of VL have been reported in northern Ethiopia, which can also involve the skin [27, 28], it is unlikely that patients in our study had VL as basic clinical investigations did not raise any VL suspicion. Future studies should conduct a full clinical examination and VL diagnostic tests.

The *L. donovani* CL cases had multiple or large lesions, and among the patients from which the outcomes were described in the chart, treatment response seemed poor. Moreover, three out of four patients could not be diagnosed with microscopy. Although this is based on very limited routine data and diagnostics and treatments generally perform poorly in Ethiopia [6, 15, 29], evidence from *L. donovani* CL in Sri Lanka shows that lesions are severe, hard to diagnose by microscopy and respond poorly to SSG treatment [30]. Hence, further investigation of *L. donovani* CL patients is needed for proper patient management.

The *L. donovani* patients in our study all originated from the northwest, which is also the area that reports 80% of the VL cases in the country [2]. However, the samples in our study from the south were all from the highlands; therefore, occurrence of *L. donovani* CL in the south cannot be ruled out [31, 32].

Two of the *L. donovani* patients originated from districts known as VL-endemic areas, while the other two lived in the mid-highlands with a wide range in altitude. There are areas in the mid-highlands in northwest Ethiopia from where both CL and VL patients present in the treatment centers (previous unpublished study records). *Phlebotomus orientalis*, the vector for *L. donovani* in the lowlands, also occurs at altitudes up to 2000 m [33–35]. In such areas, coexistence of the two *Leishmania* species and the presence of the *P. orientalis* could lead to inter-species hybridization in the gut of the vector [36]. Similarly, the predominant VL population in northern Ethiopia is migrant workers who become infected in the lowlands [21]. Outside of the harvesting season, they travel back to the highlands where *P. pedifer* and *L. aethiopica* occur. Natural hybrids of *L. aethiopica* and *L. donovani* have been identified, with up to one third of the genetic repertoire from *L. donovani* [37, 38]. Although it was confirmed with two independent gene fragments that patients in our study were infected with *L. donovani*, ITS-1 and HSP70 are respectively short and conserved fragments. As we mostly used routine samples that were stored under unfavorable circumstances, whole-genome sequencing could not be performed to reveal genetic diversity and potential hybridization. Evidence that such events occur is relevant for containment of the emergence of leishmaniasis, but also other infectious diseases globally, to which climate change will contribute considerably in the future.

Although we expected to find *L. tropica*, the rest of the samples in our study were all *L. aethiopica*. However, as we did not receive samples from the area where *L. tropica* was previously reported as causative agent for one patient [14], we cannot draw further conclusions about *L. tropica* causing CL in Ethiopia.

Our data show that *L. aethiopica* occurs across the country. A recent study using cultured isolates from Ethiopia found evidence that *L. aethiopica* is genetically highly diverse, even within a small geographical area [38]. This should be further investigated to understand whether genomics can be the determinant for the variable clinical presentations and poor treatment outcomes.

Limitations of our study are the low number of samples for which the species could successfully be determined and lack of whole-genome sequences due to unfavorable sample types and storage conditions. Moreover, samples from hospitals in Tigray region were not available because of the conflict as well as from the eastern foci because of the limited cases reported. As most samples were collected from routine diagnosis, limited sociodemographic, clinical and treatment data were available for patients, making it hard to draw solid conclusions on transmission and manifestations of CL caused by *L. donovani*.

Future studies should aim to acquire a higher number of *L. donovani* CL patients, with detailed documentation on the possible location of infection through detailed residence and travel history, clinical presentation of patients, performance of current diagnostics and treatment outcomes. If treatment outcomes are poor, further investigation for an effective treatment should be performed. Whole-genome sequencing should be performed on freshly collected samples of such patients and should be compared to *L. donovani* derived from VL patients coming from similar areas to determine the genetic variability. Moreover, whether *P. orientalis* is a permissive vector for *L. aethiopica* and *L. donovani* should be investigated, and efforts should be made to screen sand flies and potential reservoir hosts for infection with hybrids in areas where both species occur.

## Conclusions

The majority of CL in patients in Ethiopia is caused by *L. aethiopica*, but *L. donovani* can also cause CL lesions. This could potentially have implications for patient management and disease prevention. Our findings should spark future investigations into where, how and to which extent *L. donovani* CL transmission takes place and whether cases can be diagnosed and treated with the currently available tools and drugs.

## Data Availability

The datasets used/analyzed during the current study are available on the public repository Zenodo “10.5281/zenodo.10370524”.

## Abbreviations

AMGH: Arba Minch General Hospital
APHI: Amhara Public Health Institute
BMH: Boru Meda Hospital
CL: Cutaneous leishmaniasis
Ct: Cycle threshold value
DCL: Diffuse cutaneous leishmaniasis
DNA: Deoxyribonucleic acid
EPHI: Ethiopian Public Health Institute
HIV: Human immunodeficiency virus
HRM: High-resolution melt curve
HSP: Heat shock protein
IQR: Interquartile range
ITS: Internal transcribed spacer
LCL: Localized cutaneous leishmaniasis
LRTC: Leishmaniasis Research and Treatment Center
MCL: Mucocutaneous leishmaniasis
MEGA: Molecular evolutionary genetic analysis
NTD: Neglected tropical disease
PCR: Polymerase chain reaction
RFLP: Restriction fragment length polymorphism
RNA: Ribonucleic acid
SNNPR: Southern Nations Nationalities and Peoples’ Region
SSG: Sodium stibogluconate
Tm: Melting temperature
VL: Visceral leishmaniasis
